# *Shigella* MreB promotes polar IcsA positioning for actin tail formation

**DOI:** 10.1242/jcs.226217

**Published:** 2019-05-02

**Authors:** Sina Krokowski, Sharanjeet Atwal, Damián Lobato-Márquez, Arnaud Chastanet, Rut Carballido-López, Jeanne Salje, Serge Mostowy

**Affiliations:** 1 Section of Microbiology, MRC Centre for Molecular Bacteriology and Infection, Imperial College London, Armstrong Road, London SW7 2AZ, UK; 2 Department of Immunology & Infection, London School of Hygiene & Tropical Medicine, Keppel Street, London WC1E 7HT, UK; 3 Centre for Tropical Medicine and Global Health, Nuffield Department of Medicine, University of Oxford, Oxford OX3 7JT, UK; 4 Mahidol-Oxford Tropical Medicine Research Unit, Faculty of Tropical Medicine, Mahidol University, Bangkok 10400 PHRI 07103, Thailand; 5 Public Health Research Institute, Rutgers Biomedical and Health Science, Newark, New Jersey NJ 07103, USA; 6 MICALIS Institute, INRA, AgroParisTech, Université Paris-Saclay, 78350 Jouy-en-Josas, France

**Keywords:** Actin, IcsA, MreB, Septin, *Shigella*

## Abstract

Pathogenic *Shigella* bacteria are a paradigm to address key issues of cell and infection biology. Polar localisation of the *Shigella* autotransporter protein IcsA is essential for actin tail formation, which is necessary for the bacterium to travel from cell-to-cell; yet how proteins are targeted to the bacterial cell pole is poorly understood. The bacterial actin homologue MreB has been extensively studied in broth culture using model organisms including *Escherichia coli*, *Bacillus subtilis* and *Caulobacter crescentus*, but has never been visualised in rod-shaped pathogenic bacteria during infection of host cells. Here, using single-cell analysis of intracellular *Shigella*, we discover that MreB accumulates at the cell pole of bacteria forming actin tails, where it colocalises with IcsA. Pharmacological inhibition of host cell actin polymerisation and genetic deletion of IcsA is used to show, respectively, that localisation of MreB to the cell poles precedes actin tail formation and polar localisation of IcsA. Finally, by exploiting the MreB inhibitors A22 and MP265, we demonstrate that MreB polymerisation can support actin tail formation. We conclude that *Shigella* MreB promotes polar IcsA positioning for actin tail formation, and suggest that understanding the bacterial cytoskeleton during host–pathogen interactions can inspire development of new therapeutic regimes for infection control.

This article has an associated First Person interview with the first author of the paper.

## Abstract (precis)

**Summary:** The pathogen *Shigella* forms actin tails to move through the cytosol of infected cells. We show that the bacterial actin homologue MreB can help to position the autotransporter protein IcsA for such actin tail formation.

## INTRODUCTION

*Shigella* is a Gram-negative enteroinvasive bacterium and important human pathogen leading to ∼164,000 deaths annually (Khalil et al., 2018; Kotloff et al., 2017). *Shigella flexneri* and *Escherichia coli* are closely related, but *S. flexneri* harbours a virulence plasmid encoding a type III secretion system (T3SS) to inject proteins into the host cell to promote invasion (Parsot, 2009; Sansonetti et al., 1982). Minutes after invasion, *S. flexneri* escapes the phagocytic vacuole and enters the cytosol, where it replicates and polymerises actin tails that enable bacterial dissemination from cell-to-cell (Welch and Way, 2013). *Shigella* actin-based motility relies on the bacterial autotransporter protein IcsA, which localises to the cell pole inside the bacterial cytosol with the help of DnaK (Janakiraman et al., 2009), and is secreted through the inner membrane with the help of the Sec system (Brandon et al., 2003). For localisation to the outer membrane, IcsA requires chaperone proteins DegP, Skp and SurA (Purdy et al., 2002, 2007). In the *Shigella* outer membrane, the protease IcsP (also known as SopA) (Robbins et al., 2001), lipopolysaccharide (LPS) (Sandlin et al., 1995) and cardiolipin (Rossi et al., 2017) are important to maintain polar IcsA localisation. Here, IcsA can recruit host cell neural Wiskott–Aldrich syndrome protein (N-WASP, also known as WASL) and the actin-related protein 2/3 (Arp2/3) complex to polymerise host actin to mediate its motility (Egile et al., 1999; Suzuki et al., 1998). However, to counteract actin-based motility, the septin cytoskeleton can entrap actin-polymerising *Shigella* in cage-like structures and target bacteria to the autophagy pathway (Krokowski et al., 2018; Mostowy et al., 2010; Sirianni et al., 2016), an intracellular degradation process crucial for cell autonomous immunity (Randow et al., 2013).

The bacterial cytoskeleton regulates various cellular processes crucial for development, including cell division and morphogenesis (Cabeen and Jacobs-Wagner, 2010). Although mostly performed in broth culture, rearrangement of the bacterial cytoskeleton has been the subject of intense investigation (Surovtsev and Jacobs-Wagner, 2018). Work has shown that the actin homologue MreB assembles into distinct patches moving circumferentially around the bacterial cell to organise new peptidoglycan insertion during sidewall elongation, determining rod cell shape (Dominguez-Escobar et al., 2011; Garner et al., 2011; van Teeffelen et al., 2011). In *Caulobacter crescentus*, MreB dynamics have been proposed to establish global cell polarity through asymmetric localisation of developmental regulators at the cell poles (Gitai et al., 2004). Two pioneering studies artificially producing IcsA in *E. coli* have proposed that MreB is required for the restriction of polar material (Nilsen et al., 2005; Shih et al., 2005). In this case, genetic or pharmacologic manipulation of MreB caused IcsA to localise in multiple faint patches on the bacterial surface. However, MreB has never been visualised in pathogenic bacteria during infection of host cells, and the role of MreB in IcsA positioning has not been tested *in vivo*. Here, we reveal that a subpopulation of intracellular *S. flexneri* cells remodel MreB, which helps to position IcsA at the cell pole and promotes actin tail formation.

## RESULTS AND DISCUSSION

### MreB relocalises to the cell pole of intracellular *Shigella* polymerising actin tails

**Fig. 1.**
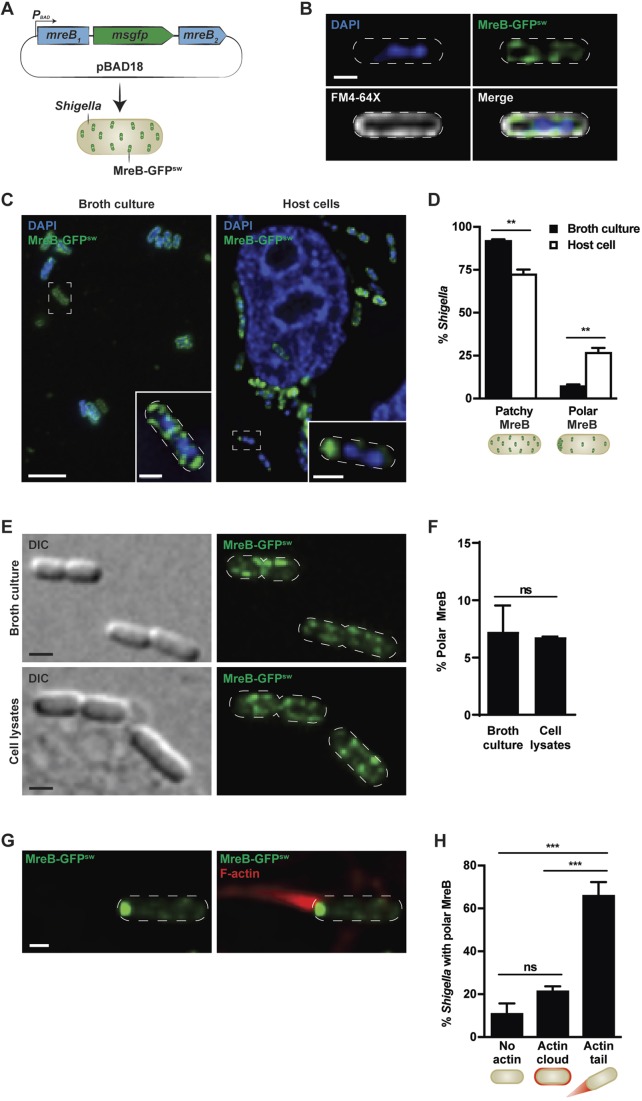
***Shigella* forming actin tails remodel MreB.** (A) Diagram illustrating the plasmid-encoded arabinose-controlled MreB-monomeric superfolder green fluorescent protein (msGFP) sandwich fusion in *S. flexneri*. (B) Localisation of MreB-GFP^sw^ in *S. flexneri* in broth culture with respect to membrane (FM4-64X) and DNA (DAPI) staining. Scale bar: 1 µm. (C) *S. flexneri* MreB-GFP^sw^ grown for 3 h in broth culture or at 3 h 40 min post infection. Scale bar: 5 µm (main image), 1 µm (inset). (D) Graph representing the mean±s.e.m. percentage of *S. flexneri* exhibiting patchy MreB-GFP^sw^ or accumulation of MreB-GFP^sw^ at the bacterial cell pole. Values are from 1107 bacteria for ‘broth culture’ and 1846 bacteria for ‘host cell’ from three independent experiments performed as in C. **P<0.01 (Student's *t*-test). (E) Representative images of *S. flexneri* MreB-GFP^sw^ grown for 2 h in broth or host cell lysates. DIC, differential interference contrast images. Scale bar: 1 µm. (F) Graph representing the mean±s.e.m. percentage of *S. flexneri* exhibiting polar MreB-GFP^sw^ accumulation. Values are from 996 bacteria for ‘broth culture’ and 933 bacteria for ‘cell lysates’ from three independent experiments performed as in E. ns, not significant, *P*>0.05 (Student's *t*-test). (G) Representative images of *S. flexneri* MreB-GFP^sw^ polymerising an actin tail. HeLa cells were infected with *S. flexneri* MreB-GFP^sw^ for 2 h 40 min and labelled for F-actin. Scale bar: 1 µm. (H) Graph representing mean±s.e.m. percentage of polar *S. flexneri* MreB-GFP^sw^ that do not polymerise actin, polymerise an actin cloud or polymerise an actin tail. Values are from 1346 bacteria from three independent experiments performed as in E., ns, not significant, *P*>0.05; ***P<0.001 (one-way ANOVA). The white dashed lines in B, the inset in C, and in E and G indicate the bacterial cell edge.

We engineered *S. flexneri* M90T bearing a plasmid-encoded inducible MreB-GFP^sw^ (internal msGFP sandwich) fusion to enable us to visualise MreB during infection of host cells (Fig. 1A,B). Considering that MreB-GFP^sw^ is functional in *E. coli* (Ouzounov et al., 2016), and that the protein sequence of *E. coli* MreB and *S. flexneri* MreB is 100% identical (Fig. S1A), we reasoned that MreB-GFP^sw^ would also be functional in *Shigella*. In agreement with this, production of MreB-GFP^sw^ did not affect *Shigella* cell dimensions, growth or intracellular viability during infection, indicating that it does not perturb cell physiology (Fig. S1B–D). Quantitative microscopy showed that for 92.3±0.5% (mean±s.e.m.) of *Shigella* cells vegetatively growing in broth culture, MreB-GFP^sw^ forms distinct patches along the cell cylinder (Fig. 1C,D), in agreement with the subcellular localisation of MreB-GFP^sw^ in *E. coli* (Ouzounov et al., 2016). Next, to follow MreB in intracellular bacteria, we infected human epithelial HeLa cells with *S. flexneri* MreB-GFP^sw^ for 2 h 40 min or 3 h 40 min. In contrast to what is seen for bacteria growing in broth culture, we found that a subpopulation of intracellular *Shigella* (18.4±2.1% or 27.2±2.4%, respectively) presents an accumulation of MreB at one bacterial cell pole (Fig. 1C,D; Fig. S1E). In these cells, MreB is observed as a single bright polar spot (in addition to being observed as faint patches along the sidewall). These results suggest that a subpopulation of intracellular *Shigella* remodels MreB during infection. To test whether we could mimic intracellular conditions that induce the polar accumulation of MreB in bacteria, we cultured *Shigella* MreB-GFP^sw^ in broth or purified HeLa cell-free extracts. Here, we found that cell-free extracts fail to induce polar accumulation of MreB (Fig. 1E,F). These results are in agreement with studies showing that wild-type *Shigella* do not polymerise actin in *Xenopus laevis* extracts because levels of IcsA are low *in vitro* (Magdalena and Goldberg, 2002). We conclude that molecular signals (host and/or bacterial) triggered during infection of host cells are required for polar accumulation of MreB.

Considering that previous work using HeLa cells showed that ∼24% of intracellular *Shigella* form actin tails at 1 h 40 min post infection (Mostowy et al., 2010), we wondered whether polar accumulation of MreB correlates with actin tail formation. To test this, we labelled *Shigella* MreB-GFP^sw^-infected cells for F-actin and found that 88.1±4.2% of *Shigella* exhibiting polar accumulation of MreB also polymerise actin, either as actin clouds or actin tails. Moreover, this subpopulation is significantly more (3.1±0.5 fold) associated with actin tails rather than actin clouds (Fig. 1G,H). These results suggest that MreB accumulation at the cell pole of intracellular *Shigella* correlates with actin tail formation.

### MreB positions IcsA at the bacterial cell pole

**Fig. 2.**
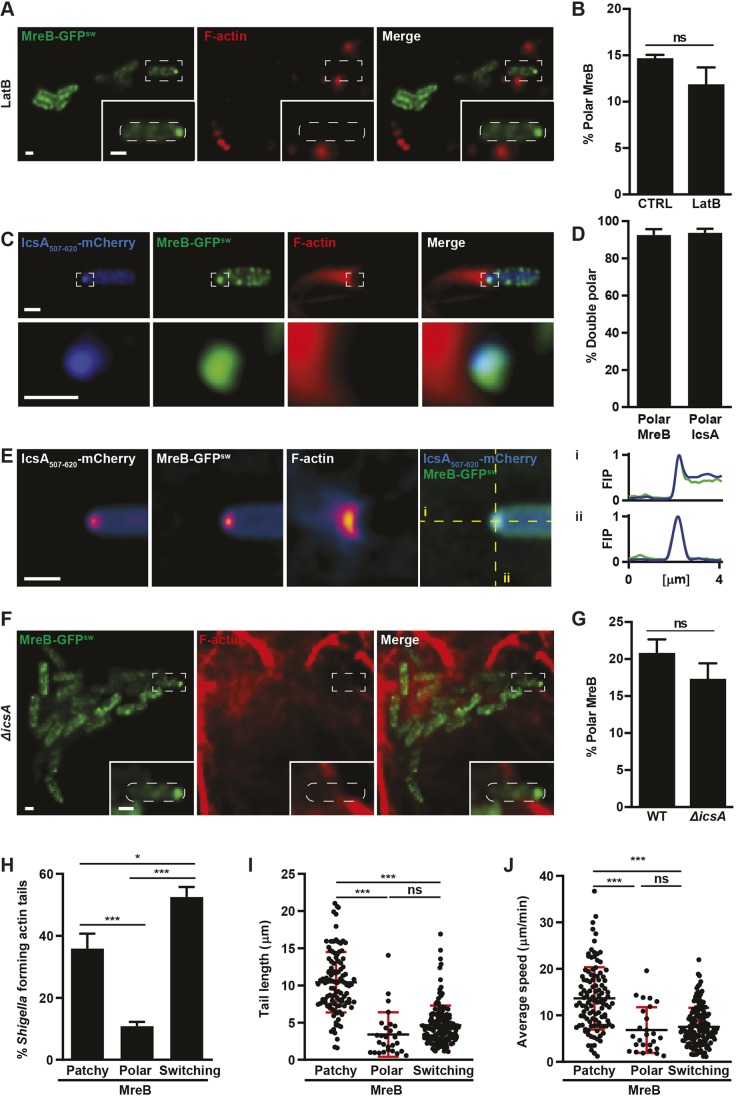
**MreB and IcsA colocalise at the same bacterial cell pole.** (A) HeLa cells were infected with *S. flexneri* MreB-GFP^sw^ for 1 h 40 min, treated with LatB for 60 min and labelled for F-actin. Scale bars: 1 µm. (B) Graph representing the mean±s.e.m. percentage of *S. flexneri* exhibiting polar MreB-GFP^sw^ localisation in untreated (CTRL) or LatB-treated conditions. Values are from 2284 bacteria for CTRL and 1463 bacteria for LatB from three independent experiments performed as in A. ns, not significant, *P*>0.05 (Student's *t*-test). (C) Representative Airyscan image of HeLa cells infected for 2 h 40 min with *S. flexneri* MreB-GFP^sw^ IcsA_507-620_-mCherry and labelled for F-actin. Scale bar: 1 µm. (D) Graph representing the mean±s.e.m. percentage of bacteria with polar MreB-GFP^sw^ that also have polar IcsA_507-620_-mCherry and vice versa. Values are from *n*=1541 bacteria from three independent experiments performed as in C. (E) Airyscan-SPA of bacteria exhibiting polar MreB accumulation, and resulting models for IcsA_507-620_-mCherry and F-actin from *n*=70 bacteria. Scale bar: 1 µm. Fluorescence intensity profiles (FIP) along the long (i) and short (ii) axis of the cell are shown to the right. Yellow dashed lines indicate where the FIPs were taken. (F) Representative image of HeLa cells infected with *S. flexneri* Δ*icsA* MreB-GFP^sw^ for 2 h 40 min. Scale bars: 1 µm. (G) Graph representing the mean±s.e.m. percentage of wild-type (WT) or Δ*icsA S. flexneri* exhibiting polar MreB-GFP^sw^ localisation. Values are from 1622 bacteria for WT and 1634 bacteria for Δ*icsA* from four independent experiments performed as in F. ns, not significant, *P*>0.05 (Student's *t*-test). (H) Graph representing mean±s.e.m. percentage of *S. flexneri* forming actin tails exhibiting patchy or polar MreB-GFP^sw^ localisation or switching between patchy and polar MreB-GFP^sw^ localisation during the imaging period. Values are from 275 bacteria from five independent experiments performed as in H. *P<0.05, ***P<0.001 (Student's *t*-test). (I,J) Graph representing mean±s.d. actin tail length or average speed of actin-polymerising bacteria exhibiting patchy, polar or switching MreB-GFP^sw^. Each dot represents a single bacterium from five independent experiments performed as in H. ns, not significant, *P*>0.05; ***P<0.001 (Student's *t*-test). The white dashed lines in the insets indicate the bacterial cell edge.

Does actin tail formation cause MreB to accumulate at the bacterial cell pole? To investigate this, we treated *Shigella* MreB-GFP^sw^-infected cells with Latrunculin B (LatB), an inhibitor of eukaryotic actin polymerisation. Fluorescence microscopy showed that LatB-treated cells are rounded and without actin stress fibres (Fig. S2A). Under these conditions, actin tails did not form (Fig. S2B), and MreB localised to the bacterial cell pole as often as in untreated conditions (Fig. 2A,B). These data show that the polar localisation of MreB does not depend on the presence of polymerised actin, suggesting that localisation of MreB can precede actin tail formation.

The bacterial autotransporter protein IcsA also localises to the *Shigella* cell pole before actin tail formation. Therefore, we investigated whether polar MreB colocalises with IcsA. We infected cells with *Shigella* simultaneously producing MreB-GFP^sw^ and IcsA_507-620_-mCherry [a derivative of IcsA that remains cytosolic (Nilsen et al., 2005)] and performed quantitative Airyscan confocal microscopy. From analysis of 900 bacterial cells, we found that the vast majority (>95%) of *Shigella* cell poles that accumulate MreB also accumulate IcsA, and vice versa (Fig. 2C,D). In line with this, single-particle averaging (SPA) of 70 bacterial cells from Airyscan confocal images revealed that IcsA colocalised with MreB as a ∼0.1 µm circular spot at the cell pole for bacteria polymerising actin tails (Fig. 2E).

To investigate the hierarchy of MreB and IcsA accumulation, we produced MreB-GFP^sw^ in *Shigella* Δ*icsA* cells, which are unable to polymerise actin (Bernardini et al., 1989). During infection of HeLa cells, we found that MreB-GFP^sw^ localised to the bacterial cell pole in the absence of IcsA as often as in the presence of IcsA (Fig. 2F,G). These results show that the localisation MreB to the cell pole precedes the polar localisation of IcsA, and indicates a role for MreB in cytosolic positioning of IcsA. Many other virulence factors of rod-shaped pathogenic bacteria localise to the cell pole, including proteins important for protein secretion and adhesion-prominent examples include the secretion systems of *Vibrio cholera* (Scott et al., 2001) and *Legionella pneumophila* (Conover et al., 2003), and the type IV pilus of *Pseudomonas aeruginosa* (Cowles and Gitai, 2010). It will thus be of great interest to investigate a possible conserved role for MreB in the positioning of virulence factors.

To follow the localisation of MreB during actin-based motility, we infected HeLa cells stably producing LifeAct-iRFP670 (to visualise F-actin) with *Shigella* MreB-GFP^sw^ for 2 h 10 min and performed time-lapse Airyscan Fast confocal microscopy. Here, we found that for 52.6±3.2% of bacteria polymerising actin tails, MreB can switch between accumulating at the cell pole and localising into patches along the cell cylinder (Fig. 2H). These data suggest that MreB can rearrange back into patches after positioning IcsA for the induction of actin tail formation. To test this, we determined the length and speed of actin tails formed by bacteria that exhibit polar MreB (exclusively), patchy MreB (exclusively) or switching MreB (polar and patchy) during the imaging period. Here, we found that bacteria that exhibit patchy MreB have significantly longer actin tails and move faster, as compared to bacteria that exhibit polar MreB and switching MreB (Fig. 2I,J). In the outer membrane, IcsA cleavage by the protease IcsP is well known to influence actin-based motility (Shere et al., 1997). We speculate that once actin tails are formed, MreB switching can help to replace IcsA at the cell pole after IcsA in the outer membrane has been cleaved by IcsP. Taken together, these data support a model in which polar accumulation of MreB helps to position IcsA to initiate actin tail formation but is not strictly required during actin-based motility.

### MreB polymerisation promotes *Shigella* actin tail formation

**Fig. 3.**
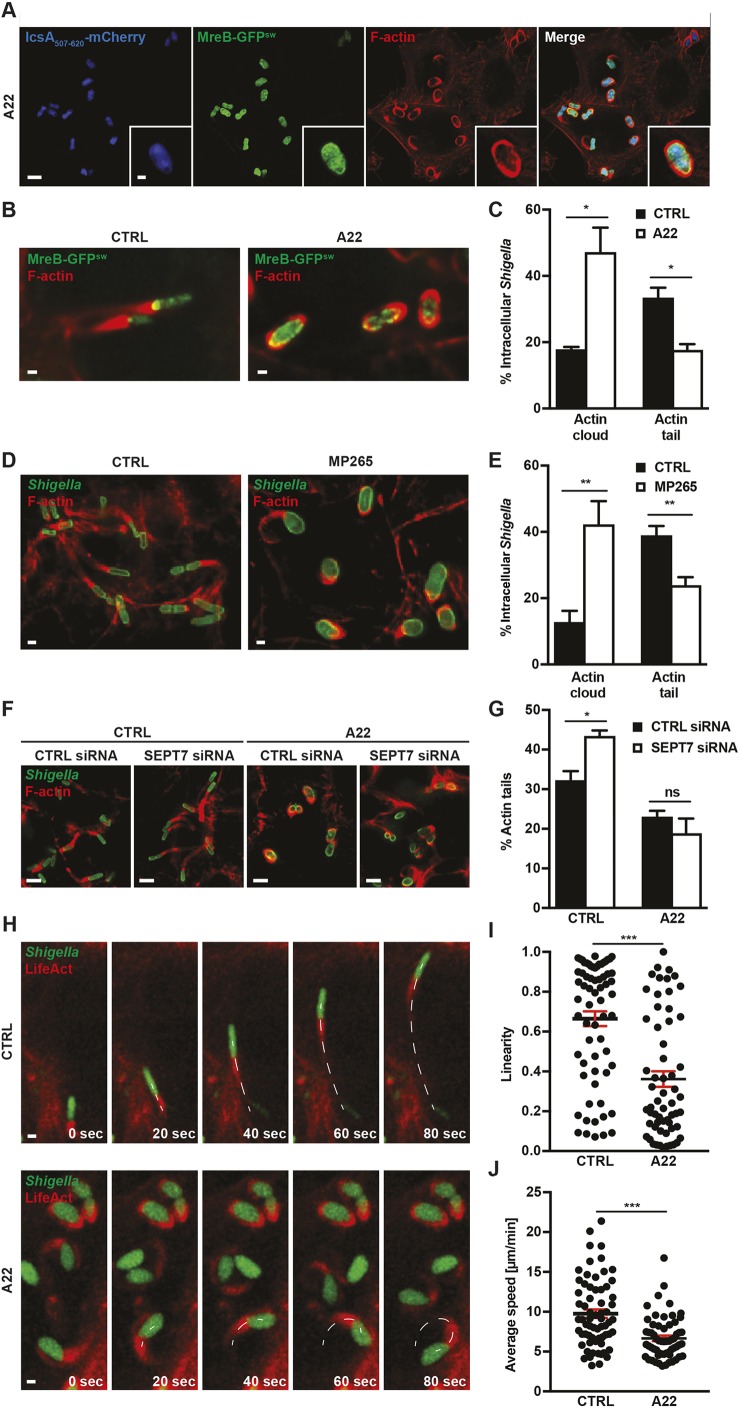
**MreB polarisation promotes *Shigella* actin tail formation.** (A) Airyscan image of HeLa cells infected for 40 min with *S. flexneri* MreB-GFP^sw^ IcsA_507-620_-mCherry, treated for 2 h with A22 and labelled for F-actin. Scale bar: 5 µm (main images), 1 µm (insets). (B) *S. flexneri* MreB-GFP^sw^ polymerising actin in untreated (CTRL) and A22-treated conditions. HeLa cells were infected for 40 min, kept untreated or treated with A22 for 2 h and labelled for F-actin. Scale bars: 1 µm. (C) Graph representing the mean±s.e.m. percentage of *S. flexneri* polymerising an actin cloud or actin tail in untreated and A22-treated conditions. Values are from 1408 bacteria for CTRL and 654 bacteria for A22 from three independent experiments performed as in B. *P<0.05 (Student's *t*-test). (D) HeLa cells were infected with *S. flexneri* for 40 min, and kept untreated (CTRL) or treated with MP265 for 2 h. They were then labelled for F-actin using Alexa Fluor 555–phalloidin and immunolabelled for *Shigella*. Scale bars: 1 µm. (E) Graph representing the mean±s.e.m. percentage of *S. flexneri* polymerising an actin cloud or actin tail in CTRL and MP265-treated conditions. Values are from 1677 bacteria for CTRL and 1614 bacteria for MP265 from four independent experiments performed as in D. **P<0.01 (Student's *t*-test). (F) HeLa cells treated with control (CTRL) or SEPT7 siRNA, infected with *S. flexneri* for 40 min and kept untreated (CTRL) or treated with A22 for 2 h. Scale bars: 5 µm. (G) Graph representing the mean±s.e.m. percentage of *S. flexneri* polymerising actin tails in HeLa cells treated with control (CTRL) siRNA or SEPT7 siRNA and kept untreated (CTRL) or treated with A22. Values are from 932 bacteria for CTRL siRNA and CTRL, 852 bacteria for CTRL siRNA and A22, 941 bacteria for SEPT7 siRNA and CTRL and 1002 bacteria for SEPT7 siRNA and A22 from three independent experiments performed as in F. ns, not significant, *P*>0.05; *P<0.05 (Student's *t*-test). (H–J) Time-lapse images (H) and quantifications (I,J) of HeLa cells transfected with LifeAct-mCherry and infected with *S. flexneri* GFP at 2 h 40 min post infection imaged every 10 s in untreated (CTRL) and A22-treated conditions. Images are cropped from Movies 1 and 2. The white dotted line indicates the bacterial trajectory. Scale bars: 1 µm. Each dot represents the linearity (I) or average speed (J) of a bacterium polymerising actin.

To investigate whether *Shigella* can form actin tails in the absence of MreB polymerisation, we used the MreB inhibitor S-(3,4-dichlorobenzyl)isothiourea (A22), an antibiotic-like small molecule that prevents MreB polymerisation and leads to coccoid bacterial cells (Fig. S3A,B). When added to infected cells, intracellular bacteria became coccoid (Fig. S3C). A22 did not affect host actin polymerisation or cell viability (Fig. S3D–F); bacterial viability is also not affected by 2 h of A22 treatment (Fig. S3G,H). Airyscan confocal imaging of MreB and IcsA confirmed that both proteins are diffusely localised in the cytosol of bacteria inside A22-treated cells (Fig. 3A). Strikingly, actin tail formation was perturbed in A22-treated conditions, leading to significantly less (1.9±0.2 fold) actin tails and significantly more (2.6±0.3 fold) actin clouds (Fig. 3B,C). Similar results were obtained using the MreB inhibitor MP265, a structural analogue of A22 (Fig. 3D,E). Taken together, these data reveal that inhibition of MreB polymerisation prevents IcsA recruitment at the cell pole and reduces actin tail formation.

Septin cage entrapment reduces *Shigella* actin tail formation (Mostowy et al., 2010, 2011), raising the possibility that A22-treated bacteria form fewer actin tails due to more septin cage entrapment. To investigate this, we treated *Shigella*-infected cells with control or SEPT7 siRNA (Fig. S3I). Similar to results for cells depleted for SEPT2 or SEPT9 (Mostowy et al., 2010), we found significantly more (1.4±0.1 fold) actin tails in SEPT7-depleted cells than in control cells (Fig. 3F,G). When siRNA-treated cells were treated with A22, we observed the same amount of actin tails in SEPT7-depleted cells as in control cells (Fig. 3F,G). These data demonstrate that A22-treated bacteria are not entrapped in septin cages to a greater extent than control cells, suggesting that their defect in actin tail formation is due to inhibition of MreB polymerisation.

To explore the role of MreB polymerisation in actin-based motility, we performed time-lapse microscopy using LifeAct-mCherry-transfected HeLa cells infected with *Shigella* expressing a soluble GFP in untreated or A22-treated conditions. In this case, movement of the bacteria (i.e. both the linearity and speed of *Shigella* cells) is significantly reduced in the presence of A22 (Fig. 3H–J; Movies 1 and 2). Therefore, we conclude that MreB polymerisation can promote IcsA polarisation, actin tail formation and efficient actin-based motility.

### Conclusion

The host cytoskeleton plays a crucial role in cell autonomous immunity and offers great therapeutic potential for infection control (Mostowy and Shenoy, 2015). Considering our discovery that MreB promotes actin tail formation, MreB can also be viewed as a promising target for antimicrobials. This reported biology should encourage further work to exploit the cytoskeleton to treat bacterial infection.

## MATERIALS AND METHODS

### Bacterial strains and growth conditions

Bacterial strains used in this study are found in Table S1. *E. coli* DH5-α were grown on Lysogeny Broth (LB) agar and single colonies were selected and grown in LB broth at 37°C. *Shigella* strains were grown on trypticase soy (TCS) agar containing 0.01% (w/v) Congo Red dye at 37°C. Single Congo Red binding colonies were selected for experiments. The following selection markers were used at the indicated concentrations: carbenicillin (100 µg/ml) and chloramphenicol (30 µg/ml).

### Cell lines

HeLa (ATCC CCL) or HeLa LifeAct-iRFP670 cells (kindly provided by Michael Way, The Francis Crick Institute, London, UK; Snetkov et al., 2016) were cultured in Dulbecco's modified Eagle's medium (DMEM; GIBCO) supplemented with 10% fetal bovine serum (FBS) at 37°C and 5% CO_2_. For selection, 10 µg/ml hygromycin was added to the culturing medium of HeLa LifeAct-iRFP670 cells. Monthly checks for mycoplasma contamination and other bacterial infections were performed.

### Measuring bacterial growth

*Shigella* strains (wild-type, pBAD18 and MreB-GFP^sw^) were grown in TCS broth overnight and diluted in TCS the next day to a starting optical density at 600 nm (OD_600_) of 0.01. Samples were prepared in triplicates in a 96-well plate. OD_600_ was measured every 30 min for 10 h at 37°C with shaking using a microplate reader (TECAN Infinite M200 Pro).

### Construction of plasmids

Constructed plasmids were sequenced at Genewiz (South Plainfield, New Jersey). Primers and plasmids used in this study are listed in Table S1. MreB-GFP^sw^ was engineered in pSA10 (IPTG-induced expression) using Advanced Quick Assembly (AQUA) cloning (Beyer et al., 2015). The sandwich fusion consists of the GFP sequence flanked by short in-frame linkers inserted between *mreB*_1-684_ and *mreB*_685-1044_. MreB was amplified from *S. flexneri* M90 T chromosomal DNA using primers SK-67 (fw) and SK-4 (rev) for *mreB*_1-684_ and SK-5 (fw) and SK-74 (rev) for *mreB*_685-1044_. The monomeric superfolder GFP was amplified from plasmid pDHL584 using primers SK-3 (fw) and SK-6 (rev) and pSA10 was amplified using primers SK-69 and SK-76. PCR products were gel purified by using a QIAquick PCR purification kit (Qiagen), mixed in equimolar amounts and incubated in milliQ water for 1 h at room temperature. Following this, chemically competent *E. coli* were transformed with the AQUA mix, and clones were screened for the correct assembly by performing colony PCR. Owing to leakage of the IPTG-controlled promoter in pSA10, MreB-GFP^sw^ was amplified using SK-67 (fw) and SK-74 (rev) and enzymatically transferred into pBAD18 using EcoRI and SalI. Finally, pBAD18_MreB-GFP^sw^ was electroporated into *S. flexneri*. Production of MreB-GFP^sw^ was suppressed by adding 1% glucose and induced by adding 0.2% arabinose to the culture medium.

### Pharmacological inhibition

For experiments involving pharmacological inhibitors, HeLa cells were infected for 40 min followed by treatment with the inhibitor or the corresponding solvent (control) for 2 h prior to fixation or time-lapse microscopy. A22 was used at 4 µg/ml and MP265 was used at 1 μg/ml. To inhibit actin polymerisation, HeLa cells were treated with 5 µM LatB for 1 h prior to fixation.

### siRNA and DNA transfection

For siRNA transfection, HeLa cells were plated in six-well plates (Thermo Scientific) at 0.8×10^5^ cells per well and transfected the following day with control siRNA (Thermo Scientific AM4635) or predesigned SEPT7 siRNA (Thermo Scientific s2753) using Oligofectamine (Thermo Scientific) for 72 h. For DNA transfection, 5×10^5^ HeLa cells were seeded per MatTek glass-bottom dish (MatTek corporation) including DNA and JetPEI (Polyplus transfection), and were used 24 h later.

### Antibodies

Rabbit polyclonal antibody used was anti-SEPT7 (IBL 18991, 1:500). Mouse monoclonal antibody used was GAPDH (AbCam ab8245, 1:500). Horseradish peroxidase-conjugated secondary antibodies used were goat anti-rabbit-IgG (Dako P0448, 1:2000) or goat anti-mouse-IgG (Dako P0260, 1:2000). F-actin was labelled with Alexa Fluor 488- or Alexa Fluor 555-conjugated phalloidin (Molecular Probes A12379 or A34055, 1:100).

### Measuring cell death

HeLa cell death was quantified after treatment with 0 (CTRL), 4 or 10 μg/ml A22 for 1 h or 2 h using 0.2% Trypan Blue and a Neubauer cell chamber. To follow cell death over time, 10^4^ HeLa cells were seeded in 96-well plates (Thermo Scientific) and used for experiments 24 h later. Samples were kept untreated or were treated with 10 μg/ml A22, and 0.05 μM SYTOX Orange nucleic acid stain (Invitrogen) was added. Emission (535–595 nm) was recorded hourly for 12 h using a microplate reader (TECAN Infinite M200 Pro).

### Incubation of bacterial cells with host cell lysates

HeLa cells were washed twice with PBS and once in lysate buffer (50 mM Tris-HCl pH 8, 50 mM KCl, 0.5 mM MgCl_2_, 1 mM DTT, complete protease inhibitor cocktail, 0.1 mM PMSF and 0.1% BSA). Cells were lysed with 30–40 strokes with a homogeniser, and cell lysis was confirmed by using a light microscope. Bacterial cells were cultured in TCS broth overnight at 37°C and diluted 50× in TCS the following day. *Shigella* were grown to an OD_600nm_ of 0.4 and washed two times in TCS (CTRL) or lysate buffer. Samples were diluted to a starting OD_600nm_ of 0.1 and 100 µl culture was centrifuged for 3 min at 5939 ***g***. Bacteria were resuspended in equal volumes of TCS (CTRL) or host cell lysates and incubated for 2 h at 37°C while shaking. Finally, 1.5 µl bacterial culture was placed on 2% low-melting point agarose pads for epifluorescence microscopy.

### Bacterial infection of epithelial cells

HeLa (ATCC CCL) cells were cultured in DMEM (GIBCO) supplemented with 10% fetal bovine serum (FBS) at 37°C and 5% CO_2_. Cells for fixed microscopy were plated (10^5^) on glass coverslips in six-well plates (Thermo Scientific) and used for experiments 48 h later. HeLa cells for time-lapse microscopy were grown (5×10^5^) on MatTek glass-bottom dishes (MatTek corporation) and infected 24 h later.

For infection assays, *Shigella* were grown in TCS broth overnight at 37°C and diluted 50× in TCS the following day. Bacteria were cultured until they reached an OD_600nm_ of 0.4–0.7. *Shigella* were diluted in DMEM and added to HeLa cells at a multiplicity of infection (MOI) of 100. Samples were centrifuged at 110 ***g*** for 10 min at 21°C and were incubated for 30 min at 37°C and 5% CO_2_. To remove extracellular bacteria, cells were washed three times with PBS and incubated in fresh, 50 μg/ml gentamicin-containing medium for 1 h, 1 h 30 min, 2 h or 3 h. MreB-GFP^sw^ production was induced with 0.2% arabinose at 40 min post infection for the remaining infection process. IcsA_507-620_-mCherry, a cytosolic derivate of IcsA that contains the region 2 targeting sequence for polar localisation (Charles et al., 2001; Nilsen et al., 2005; a kind gift from Marcia Goldberg, Harvard Medical School, Boston, MA), production was induced with 1 mM IPTG 15 min prior to fixation.

### Gentamicin protection assays

HeLa cells were grown and infected with *Shigella* as described above. Intracellular bacteria were extracted after 1 h and 4 h 40 min from infected HeLa cells by washing with PBS and lysing the cells for 5 min with Triton-X-100 at room temperature. Cell lysates were serially diluted and plated on LB agar plates and incubated at 37°C overnight. The number of colony forming units (CFU) was determined.

### Fluorescence microscopy of infected cells

To process samples for fixed microscopy, infected HeLa cells were washed with PBS and fixed in 4% paraformaldehyde (PFA) for 15 min at room temperature, before subsequently being washed with PBS and quenched for 10 min in 0.05 M ammonium chloride at room temperature. Afterwards cells were permeabilised for 5 min with 0.1% Triton-X-100 at room temperature and stained for fluorescence microscopy. Incubation with primary antibodies was performed in PBS for 1 h 30 min at room temperature or overnight at 4°C and incubation with secondary antibodies and phalloidin was performed in PBS for 45 min at room temperature. Finally, cells were incubated for 10 min in 1 μg/ml DAPI and mounted in Aqua polymount mounting medium (Polyscience Inc.). Fixed cells were imaged using a 63×/1.4 C-Plan Apo oil immersion lens on a fluorescence-inverted microscope Axiovert Z1 driven by ZEN software (Carl Zeiss) or on an LSM 880 (Carl Zeiss) in Airyscan super resolution (SR) mode driven by ZEN Black software.

For time-lapse microscopy of infected HeLa cells, samples were imaged every 15 s in FluoroBrite (A1896701, Thermo Fisher Scientific) from 2 h 10 min after infection using a 63×/1.4 C-Plan Apo oil immersion lens on an LSM 880 (Carl Zeiss) in Airyscan Fast SR mode driven by ZEN Black software (Carl Zeiss) (Fig. 2H–J) or samples were imaged every 10 s in FluoroBrite containing 50 μg/ml gentamicin from 1 h 40 min after infection using a 63×/1.4 C-Plan Apo oil immersion lens on a confocal microscope LSM 710 (Carl Zeiss) driven by ZEN 2010 software (Fig. 3H-J).

### Microscopy of bacterial cells grown in broth culture

To measure bacterial cell length and width, wild-type *Shigella* and MreB-GFP^sw^ were grown to early exponential phase and 0.1% L-arabinose was added for a further 2 h. Where indicated, *Shigella* were treated with 3 μg/ml FM4-64X (Thermo Scientific) for 30 min and/or 1 μg/ml DAPI for 10 min before analysis. Bacteria were washed in PBS and 2.5 μl bacteria solution was applied on poly-L-lysine-coated coverslips.

### Microscopy analysis

Microscopy images were quantified from *z*-stack image series by taking 8 to 15 images over 0.2–0.4 μm. Image processing was performed using Fiji (ImageJ) or Icy (http://icy.bioimageanalysis.org) and deconvolution was performed using Huygens deconvolution software or ZEN software. Bacterial cell length and width were measured manually using the plugin ‘Coli-inspector’ for FIJI. The movement (i.e. linearity and speed) of bacteria polymerizing actin were measured using the motion profiler from Icy. For single-particle analysis (Gray et al., 2016), the MreB-positive pole and long axis were manually selected. All bacterial cells were automatically aligned using these two references and the resulting stacks were averaged to create population-representative models. Metabolically active bacteria were quantified according to Sirianni et al. (2016).

### Statistics

Statistical analysis was performed in Excel (Microsoft) and Prism Graphpad. Host cells dying from bacterial load were excluded from analysis. In experiments using Δ*icsA* MreB-GFP^sw^ *Shigella* cells, only bacteria at the host cell periphery were considered for analysis to avoid analysing overlapping bacteria. Unless otherwise indicated, data represent the mean±standard error of the mean (s.e.m.) from at least three independent experiments. The D'Agostino Pearson normality test was used to test whether data are normally distributed. A Student's *t*-test (unpaired, two-tailed) or one-way ANOVA was used to test for statistical significance, with *P*<0.05 considered as significant. Fold changes were calculated from each independent experiment and the mean±s.e.m. values are given in the text.

## Supplementary Material

Supplementary information
