# Supplementary material for: Shigella MreB promotes polar IcsA positioning for actin tail formation
Source: J Cell Sci. 2019 May 2;132(9):jcs226217. doi: 10.1242/jcs.226217 (PMC6526709; doi:10.1242/jcs.226217)
Supplement: Supplementary information [file joces-132-226217-s1.pdf]

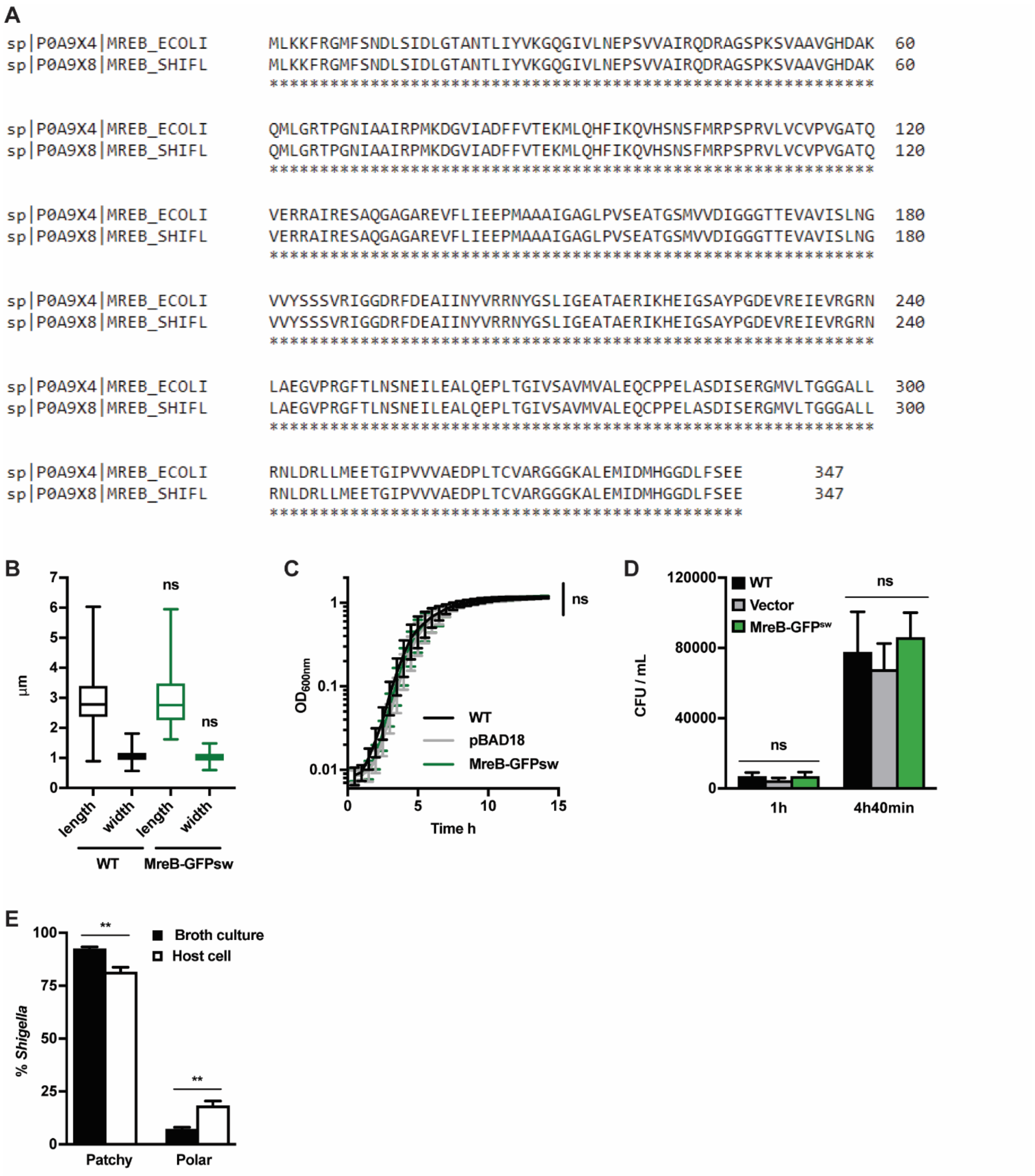

Fig. S1. ***Shigella*** **forming actin tails remodel MreB.**

(A) Sequence alignment of *E. coli* MreB (ECOLI) and *S. flexneri* MreB (SHIFL). The stars indicate 100% sequence identity.

(B) Length and width of *S. flexneri* WT and MreB-GFP^{sw}. Graph represents median and whiskers (min to max) from n = 600 bacterial cells from 3 independent experiments. Student's t-test, ns $p > 0.05$.

(C) Optical density (OD_{600}) of *S. flexneri* WT, empty pBAD18 and MreB-GFP^{sw} was measured every 30 min for 15 h using a microplate reader. Graph represents mean OD_{600} ± SEM from 7 independent experiments. Student's t-test on last time point, ns $p > 0.05$.

Journal of Cell Science • Supplementary information

(D) HeLa cells were infected with *S. flexneri* WT, empty pBAD18 or MreB-GFP^{sw} and intracellular bacterial load was determined 1 h and 4 h 40 min post infection. Invasion of host cells and intracellular survival is not affected by pBAD18 or MreB-GFP^{sw}. Graph represents mean CFU / mL ± SEM from 3 independent experiments. Student's t-test, ns $p > 0.05$.

(E) *S. flexneri* MreB-GFP^{sw} were grown for 3 h in broth culture or HeLa cells were infected with *S. flexneri* MreB-GFP^{sw} for 2 h 40 min. Graph represents mean % ± SEM of *S. flexneri* exhibiting patchy MreB-GFP^{sw} distribution or accumulation of MreB-GFP^{sw} at the bacterial pole(s). Values from 1346 bacterial cells from 3 independent experiments.

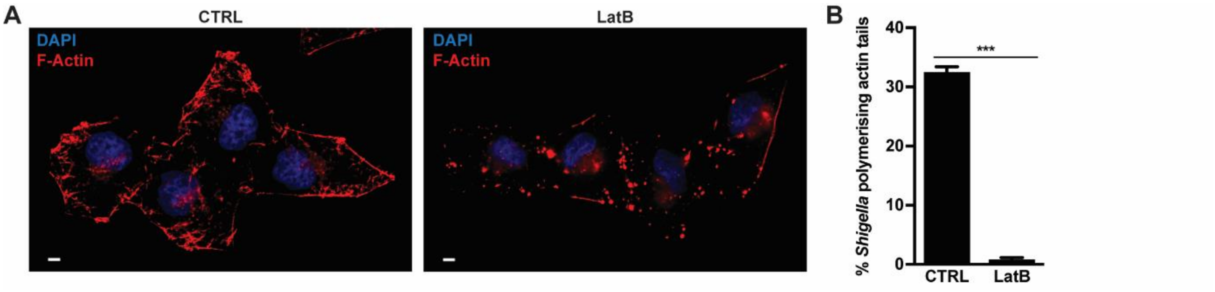

Fig. S2. MreB and IcsA colocalise at the same bacterial cell pole.

(A) HeLa cells were kept untreated or treated with Latrunculin B for 1 h and fixed and stained with 555-phalloidin to label F-actin. Scale bar = 5 µm.

(B) HeLa cells were infected with *S. flexneri* for 1 h 40 and kept untreated or treated with Latrunculin B (LatB) for 1 h further. Samples were fixed, stained for F-actin using phalloidin-555 and the amount of actin tails was quantified. Student's t-test, ***$p < 0.001$.

Journal of Cell Science • Supplementary information

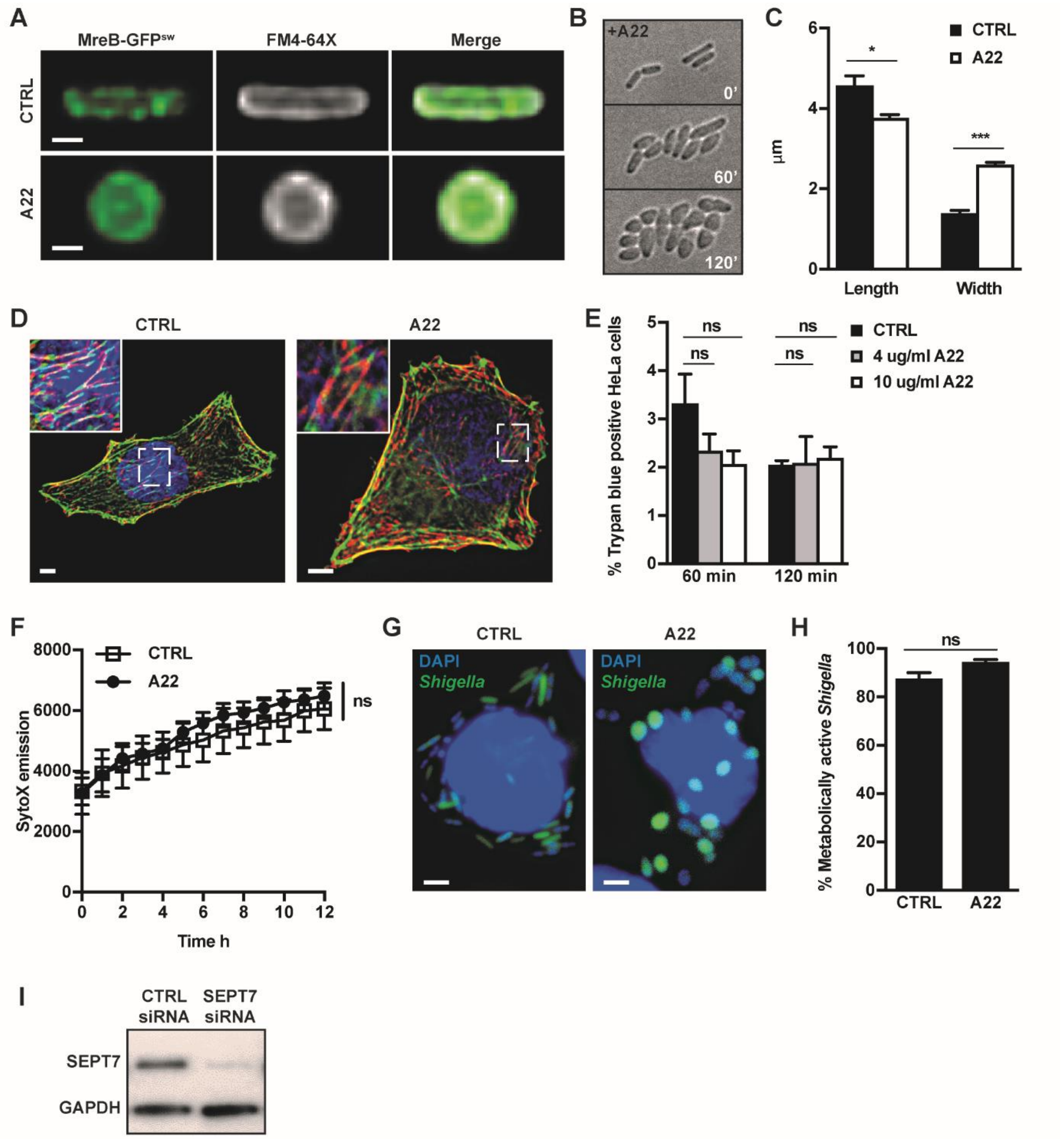

Fig. S3. MreB polarisation promotes *Shigella* actin tail formation.

(A) *S. flexneri* MreB-GFP^{sw} grown in broth culture in untreated (CTRL) or A22-treated conditions. Scale bar = 1 µm.

(B) DIC time-lapse microscopy of *Shigella* growing on agarose pads containing A22. Bacteria were imaged every 10 min for 2 h.

(C) HeLa cells were infected with *S. flexneri* for 40 min, kept untreated (CTRL) or treated with A22 for 2 h and immunolabelled for *Shigella*. Graph represents mean % ± SEM of *S. flexneri* cell length and width in CTRL or A22-treated conditions. Values from 596 bacterial cells for CTRL and 924 bacterial cells for A22 from 3 independent experiments. Student's t-test, $^{*}p< 0.05$, $^{***}p< 0.001$.

Journal of Cell Science • Supplementary information

(D) HeLa cells were kept untreated (CTRL) or treated with A22 for 2 h and labelled for F-actin (green), SEPT7 (red) and DNA (DAPI, blue). Scale bar = 5 µm.

(E) HeLa cells were kept untreated (CTRL) or treated with A22 for 2 h and the number of trypan blue positive cells was determined using a light microscope. One-way ANOVA, ns $p > 0.05$.

(F) HeLa cells were kept untreated (CTRL) or treated with A22 and the emission of the live/dead stain SYTOX was determined in a plate reader every hour for 12 h. Student’s t-test on the last time point, ns $p > 0.5$.

(G) Metabolically active (i.e. GFP producing) intracellular *Shigella* in untreated (CTRL) and A22-treated conditions. Scale bar = 5 µm.

(H) Graph represents mean % ± SEM of metabolically active *S. flexneri* in CTRL or A22-treated conditions. Values from 3111 bacterial cells for CTRL and 1473 bacterial cells for A22 from 3 independent experiments as performed in (E). Student’s t-test, ns $p > 0.05$.

(I) HeLa cells were treated with control (CTRL) or SEPT7 siRNA. Whole cell lysates of siRNA-treated cells were immunoblotted for SEPT7 or GAPDH to show SEPT7 depletion.

Table S1. List of bacterial strains, oligonucleotides and plasmids used in this study.

**Resource**	**Source**
**Bacterial Strains**	
Escherichia coli DH5α	Thermo Fisher Scientific
Shigella flexneri M90T BUG 2505 Serotype 5a	(Mostowy et al., 2010)
Shigella flexneri M90T BUG 2505 Serotype 5a GFP	(Mostowy et al., 2010)
**Oligonucleotides**	
SK-3 ACCCGGGCAGCGGTAGCAGCAGTAAAGGTGAAGAAC TGTTCACCGGTGT	This study
SK-4 TCACCTTTACTGCTGCTACCGCTGCCCGGGTAAGCCG AACCGATTTCGT	This study
SK-5 CTACAAAAGCGGTGCGCCGGGTGATGAAGTCCGTGA AATCGAAGTTCGT	This study
SK-6 GGACTTCATCACCCGGCGCACCGCTTTTGTAGAGTTC ATCCATGCCGT	This study
SK-67 AGCGGATAACAATTTCACACAGGAAACAGAATGTTGA AAAAATTTCGTGGCATGTTTTCC	This study
SK-69 ATTCCCGGGGATCCGTCGACCTGC	This study
SK-74 GGTCGACGGATCCCCGGGAATTTACTCTTCGCTGAAC AGGTCG	This study
SK-76 TCTGTTTCCTGTGTGAAATTGTTATCCGCTCACAATTCC	This study
**Plasmids**	
pBAD18	(Guzman et al., 1995)
pBAD18 MreB-GFP^{sw}	This study
pDHL584	(Landgraf et al., 2012)
pFPV25.2	(Valdivia and Falkow, 1996)
pSA10	(Schlosser-Silverman et al., 2000)
pSA10 MreB-GFP^{sw}	This study
pSA11	(Schlosser-Silverman et al., 2000)

Guzman, L. M., Belin, D., Carson, M. J. and Beckwith, J. (1995). Tight Regulation, Modulation, and High-Level Expression by Vectors Containing the Arabinose P(BAD) Promoter. *J. Bacteriol.* **177**, 4121–4130.

Landgraf, D., Okumus, B., Chien, P., Baker, T. A. and Paulsson, J. (2012). Segregation of Molecules at Cell Division Reveals Native Protein Localization. *Nat. Methods* **9**, 480–482.

Mostowy, S., Bonazzi, M., Hamon, M. A., Tham, T. N., Mallet, A., Lelek, M., Gouin, E., Demangel, C., Brosch, R., Zimmer, C., et al. (2010). Entrapment of Intracytosolic Bacteria by Septin Cage-like Structures. *Cell Host Microbe* **8**, 433–444.

Schlosser-Silverman, E., Elgrably-Weiss, M., Rosenshine, I., Kohen, R. and Altuvia, S. (2000). Characterization of Escherichia coli DNA Lesions Generated within J774 Macrophages. *J. Bacteriol.* **182**, 5225–5230.

Valdivia, R. H. and Falkow, S. (1996). Bacterial Genetics by Flow Cytometry: Rapid Isolation of Salmonella Typhimurium Acid-Inducible Promoters by Differential Fluorescence Induction. *Mol. Microbiol.* **22**, 367–378.

Journal of Cell Science • Supplementary information

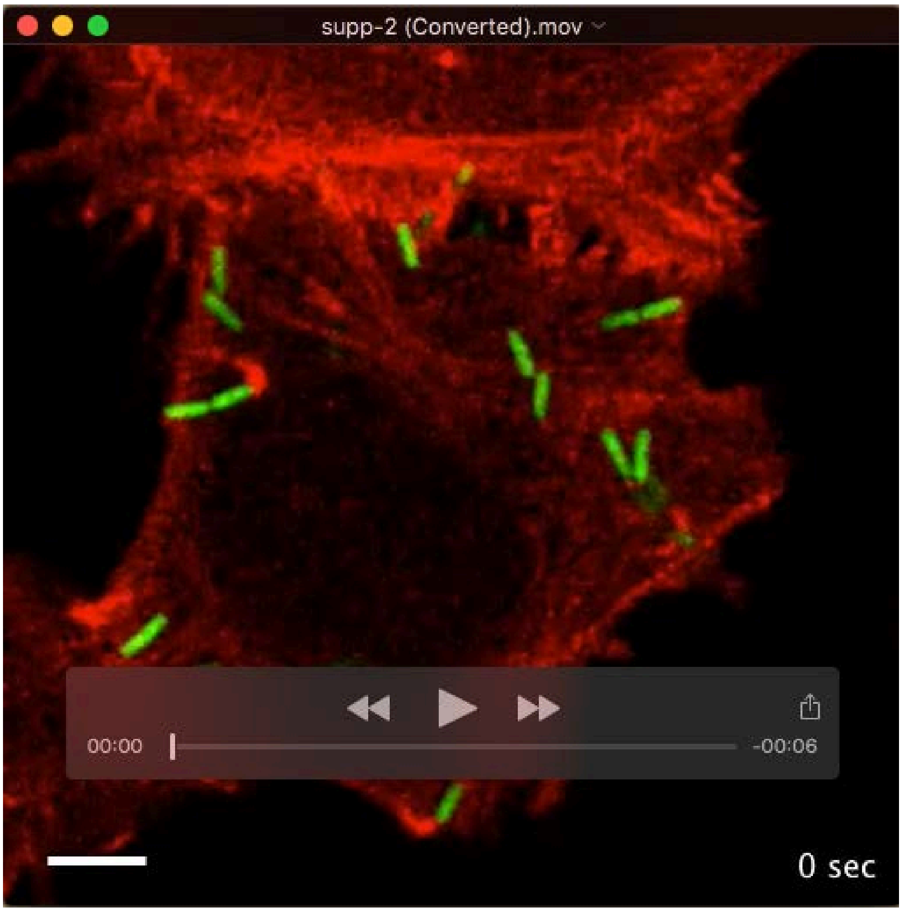

Movie 1. Actin tail in untreated conditions.

Time-lapse of HeLa cells transfected with LifeAct-mCherry and infected with *S. flexneri* GFP for 2 h 40 min. Infected cells were transferred to the microscope and imaged every 10 sec in untreated (CTRL) conditions. Scale bar = 1 µm.

Journal of Cell Science • Supplementary information

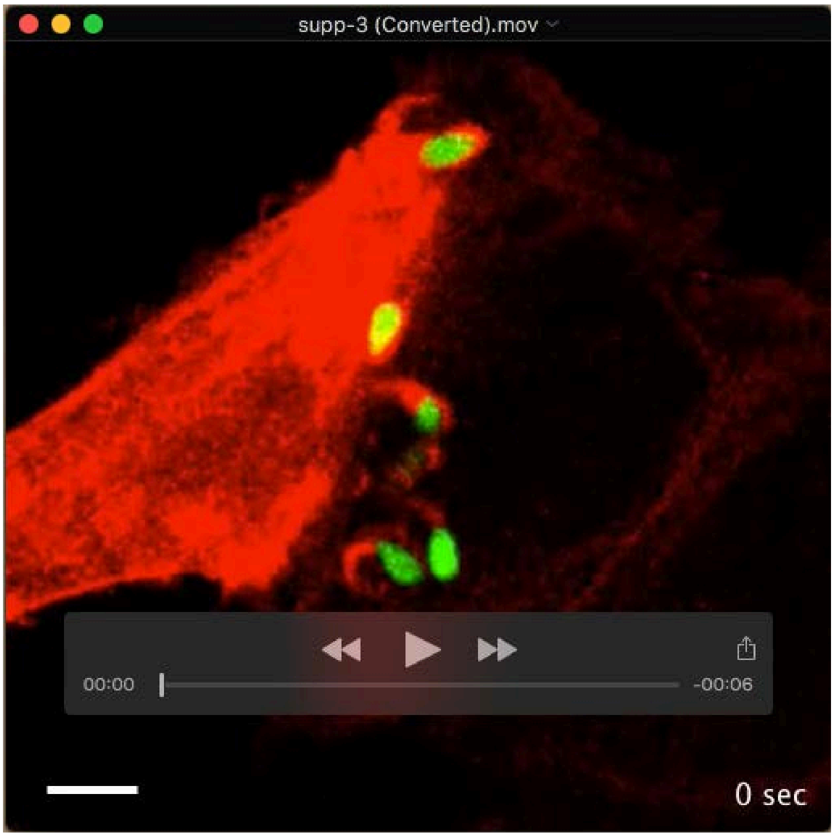

Movie 2. Actin tail in A22 conditions.

Time-lapse of HeLa cells transfected with LifeAct-mCherry and infected with *S. flexneri* GFP for 40 min and A22 was added for further 2 h. Infected cells were transferred to the microscope and imaged every 10 sec. Scale bar = 1 µm.

Journal of Cell Science • Supplementary information
